# Supplementary material for: Collaborative intelligence in AI: Evaluating the performance of a council of AIs on the USMLE
Source: PLOS Digit Health. 2025 Oct 9;4(10):e0000787. doi: 10.1371/journal.pdig.0000787 (PMC12510544; doi:10.1371/journal.pdig.0000787)
Supplement: S2 File — (ZIP) [file pdig.0000787.s002.zip › S2 Data Raw data files containing unprocessed question inputs and LLM outputs including transcripts of deliberations/STEP3_Question-129-1_Type-MC-NJ.docx]

**Facilitator** **[Round 1]:** A 39-year-old woman, gravida 6, para 5, is admitted to the hospital at 39 weeks' gestation for a scheduled induction of labor for delivery of twins. Her prior pregnancies were uncomplicated. Her current pregnancy has been complicated by well- controlled gestational diabetes. Vital signs are temperature 36.7°C (98.1°F), pulse 80/min, respirations 16/min, and blood pressure 134/84 mm Hg. Uterine size is consistent with a 36-week gestation. The fetuses are estimated to weigh 1814 g (4 lb) each. The patient delivers healthy twins. After the delivery of the placenta, examination discloses decreased uterine tone, increased bleeding, and increased hypotension. Which of the following is the most appropriate initial pharmacotherapy? (A) Ergonovine (B) Intramuscular vitamin K (C) 15-Methylprostaglandin F2α (D) Misoprostol (E) Oxytocin

**AI_D:** RESTATING THE QUESTION: The clinical scenario describes a 39-year-old woman who has just given birth to twins. Following the delivery of the placenta, she exhibits symptoms suggesting decreased uterine tone, increased bleeding, and increased hypotension. We are asked to identify the most appropriate initial pharmacotherapy to manage her condition. PLANNING: This woman presents with postpartum hemorrhage, defined as excessive bleeding after delivery. It can occur due to several reasons, including uterine atony, retained placental tissue, or coagulation disorders. The presentation, in this case, directs towards uterine atony as the primary cause, as no indication of retained placenta or coagulation complication has been given. CLINICAL REASONING: Uterine atony, a condition in which the uterus fails to contract properly after childbirth, is the leading cause of postpartum hemorrhage. Treatment usually involves medications that promote uterine contractions or "uterotonics." From the given options, Ergonovine, 15-Methylprostaglandin F2α, Misoprostol, and Oxytocin are uterotonic agents. CONCLUDING: The selection of the initial agent amongst these depends on their relative efficacy, side effect profiles, availability, and specific clinical signs. Oxytocin is often the first-line therapy for uterine atony because it is well-tolerated and effective. RESPONSE: The most appropriate initial pharmacotherapy for this patient, who shows signs of increased uterine bleeding and hypotension indicative of uterine atony, is Oxytocin (E).

**AI_B:** 1) RESTATING THE QUESTION: The query is about a woman who has just delivered healthy twins at 39 weeks. Postpartum, she presents with decreased uterine tone, increased bleeding, and hypotension. The question seeks the most appropriate first-line medication to manage this condition. 2) PLANNING: This scenario is characteristic of postpartum hemorrhage (PPH), a serious complication following childbirth that can lead to maternal death if not promptly managed. PPH, which is defined as excessive bleeding within the first 24 hours of childbirth, can have different causes with the most common being uterine atony. We are to identify a pharmacological therapy that can manage this situation effectively. Choices include ergonovine, intramuscular vitamin K, 15-Methylprostaglandin F2α, misoprostol, and oxytocin. 3) CLINICAL REASONING: Each of the listed drugs has a function in obstetric situations, but not all are indicated for PPH caused by uterine atony. Ergonovine induces uterine contractions, and so does Misoprostol and 15-Methylprostaglandin F2α. Vitamin K is primarily associated with aiding blood clotting, but it's not used as an initial therapy in PPH. Of all these options, Oxytocin is the most commonly chosen initial treatment for managing PPH caused by uterine atony. It stimulates uterine contractions and aids in shrinking the uterus back to its normal size post-delivery, thus reducing bleeding. 4) CONCLUDING: Considering the clinical evidence supporting the use of oxytocin as an initial treatment for PPH caused by uterine atony, it can be concluded that this would be the best pharmacological option for this patient. 5) RESPONSE: Based on the woman's clinical presentation and considering the common treatments for postpartum hemorrhage due to uterine atony, Oxytocin (E) would be the most appropriate initial pharmacotherapy.

**AI_A:** To rephrase the question and define some terms, we are being asked about the most suitable initial drug treatment for a 39-year-old woman, who has just given birth to twins and is experiencing hypotonia of the uterus, excess bleeding, and a drop in blood pressure following the delivery of the placenta. Hypotonia of the uterus, or uterine atony, is a condition where the uterus fails to contract following the delivery of the placenta. This failure to contract may lead to severe postpartum hemorrhage, as in this case, which can consequently cause hypotension. The clinical assumption that needs to be made here is that the hypotonic uterus, increased bleeding, and hypotension the woman is experiencing after delivery are due to postpartum hemorrhage, a common complication after childbirth. An alternate assumption could be that her symptoms are due to other complications such as retained placental tissue or coagulation disorders. However, given her symptomatology (decreased uterine tone, increased bleeding), the most clinically relevant assumption to pursue is that of postpartum hemorrhage secondary to uterine atony. The medications listed in the options all target different aspects of the postpartum period. Ergonovine is used to control bleeding by causing sustained uterine contraction, but it can cause hypertension. Misoprostol is a prostaglandin that can be used for inducing labor and preventing postpartum hemorrhage, but it's less commonly used as the first choice. 15-Methylprostaglandin F2α is used to control postpartum hemorrhage after other treatments have failed, while vitamin K is administered to the newborn to prevent hemorrhagic disease. In the context of this patient, the most critical line of treatment would be to manage her postpartum hemorrhage and uterine atony. The drug of choice in such cases is usually a uterotonic agent to enhance the contraction of the uterine muscles and control the bleeding. Oxytocin (option E) is one such agent used primarily for this condition. It triggers contractions in the uterine muscle and is often the first treatment of choice in stabilizing patients with postpartum hemorrhage due to uterine atony. Therefore my response would be to select (E) Oxytocin, as the most appropriate initial pharmacotherapy to manage this patient's condition.

**AI_E:** (1) RESTATING THE QUESTION: The woman presenting to the hospital is 39 years old and is at the full-term birthmark for her pregnancy of twins, having already had five other full-term pregnancies (gravida 6, para 5). She's also been diagnosed with gestational diabetes during this pregnancy, although it seems to be well managed. After delivering her twins and the placenta, she now displays decreased uterine tone (relaxed uterus), increased bleeding, and hypertension. We are being asked to determine the best initial medication to assist her condition. (2) PLANNING: Let's look at the medications one by one: (A) Ergonovine is a medication used to prevent postpartum hemorrhage (excessive bleeding following childbirth). (B) Intramuscular vitamin K is usually given to newborns to prevent bleeding disorders. (C) 15-Methylprostaglandin F2α is a prostaglandin used to expel products of conception post mismanaged abortion or missed abortion. (D) Misoprostol is a prostaglandin analogue used for labor induction, prevention of gastric ulcers, postpartum hemorrhage. (E) Oxytocin is a hormone that stimulates uterine contractions and milk let-down (it's given to promote labor and prevent postpartum hemorrhage). Considering the woman's situation i.e., decreased uterine tone, increased bleeding, and increased hypotension, it seems that she might be suffering from uterine atony (inability of the uterus to contract properly), which is the most common cause of postpartum hemorrhage. This would necessitate a medication that promotes uterine contractions. (3) CLINICAL REASONING: Both misoprostol and oxytocin are known to stimulate uterine contractions, which could help to reduce bleeding and eventually mitigate hypotension. Misoprostol, however, is usually associated with the risk of fever and shivering and thus is typically only used if oxytocin and ergot derivatives aren't sufficient. Considering the clinical context (uterine atony), the appropriate approach is usually to start with a first-line agent, which is Oxytocin. (4) CONCLUDING: Therefore, given the clinical signs of postpartum hemorrhage presumably due to uterine atony, Oxytocin is usually administered first due to its effectiveness and safety profile in promoting uterine contraction and thereby reducing postpartum bleeding. (5) RESPONSE: (E) Oxytocin would be the most appropriate initial pharmacotherapy for this patient.

**AI_C:** (1) RESTATING THE QUESTION: The clinical scenario given involves a 39-year-old woman, who has given birth to healthy twin babies. Post-delivery, she presents decreased uterine tone, increased bleeding, and increased hypotension. The question is asking to identify an initial pharmacological intervention that will be most appropriate for this type of presentation. (2) PLANNING: To address this question, it's important to consider the symptoms presented –decreased uterine tone and increased bleeding, indicative of a condition termed postpartum hemorrhage (PPH). PPH, a medical emergency, is often caused by uterine atony, a lack of normal muscle contraction in the uterus after delivering a baby, which can result in heavy bleeding. With this understanding, we can make the preliminary assumption that treatment should aim towards mitigating bleeding by promoting uterine contraction. As such, we should consider the pharmacological agents which have this effect. (3) CLINICAL REASONING: Considering the options given: (A) Ergonovine: It's a semi-synthetic ergot alkaloid used for the prevention of PPH by inducing sustained uterine contraction. However, it has a slow onset of action often producing unwanted substantial hypertension. (B) Intramuscular vitamin K: It has a role in blood clotting process, however, it does not directly encourage uterine contraction. (C) 15-Methylprostaglandin F2α (carboprost): It is a synthetically derived prostaglandin used to contract the uterus and reduce postpartum bleeding. However, it often reserves for cases where first-line treatments fail as it can cause bronchospasm and is contraindicated in patients with asthma. (D) Misoprostol: A synthetic prostaglandin, it promotes uterine contraction but is primarily used for medical abortion and the prevention of gastric ulcers. Its use in PPH management is considered only if other alternatives fail or aren't available. (E) Oxytocin: It's a hormone that triggers uterine contractions during labor and after delivery to reduce bleeding. As a first-line treatment, oxytocin is a rapid, effective, and well-tolerated option for preventing and managing PPH. In light of her evident uterine atony and consequent postpartum hemorrhage, the primary goal should be to augment uterine tonicity and control the bleeding. (4) CONCLUDING: Based on the clinical scenario and the pharmacological properties of the options given, oxytocin stands out as the most appropriate initial therapy. It helps in achieving adequate uterine contraction without having the significant side effects that some of the other options listed may have. (5) RESPONSE: Therefore, in this instance, the most appropriate initial pharmacotherapy for the patient experiencing symptoms of postpartum hemorrhage due to decreased uterine tone would be (E) Oxytocin.

**Facilitator** The question presents a 39-year-old woman who has just given birth to twins and is experiencing symptoms of decreased uterine tone, increased bleeding, and hypotension. These symptoms suggest a condition known as postpartum hemorrhage (PPH), often caused by uterine atony, a failure of the uterus to contract properly after childbirth. The question asks for the most suitable initial pharmacotherapy for this condition. The options provided include Ergonovine, Intramuscular vitamin K, 15-Methylprostaglandin F2α, Misoprostol, and Oxytocin. Each of these medications has a role in obstetric situations, but not all are first-line treatments for PPH caused by uterine atony. Ergonovine is used to prevent PPH by inducing sustained uterine contraction, but it can cause hypertension. Intramuscular vitamin K aids in the blood clotting process but does not directly encourage uterine contraction. 15-Methylprostaglandin F2α is used to contract the uterus and reduce postpartum bleeding, but it is often reserved for cases where first-line treatments fail. Misoprostol, a synthetic prostaglandin, promotes uterine contraction but is primarily used for medical abortion and the prevention of gastric ulcers. Its use in PPH management is considered only if other alternatives fail or aren't available. Oxytocin, on the other hand, is a hormone that triggers uterine contractions during labor and after delivery to reduce bleeding. It is a rapid, effective, and well-tolerated option for preventing and managing PPH. Given the woman's symptoms of uterine atony and consequent postpartum hemorrhage, the primary goal should be to augment uterine tonicity and control the bleeding. Therefore, Oxytocin stands out as the most appropriate initial therapy. In conclusion, based on the clinical scenario and the pharmacological properties of the options given, the most appropriate initial pharmacotherapy for this patient experiencing symptoms of postpartum hemorrhage due to decreased uterine tone would be Oxytocin (E).
